# Warranting Dental Operations

**Published:** 1860-01

**Authors:** 


### Editorial.

### WARRANTING DENTAL OPERATIONS.

The custom has obtained with a great majority of the members of the dental profession of giving an unlimited guarantee for the permanence of their operations, particularly those upon the natural teeth. Now while we consider it the duty of every operator upon the teeth, to perform all operations in as perfect manner as possible and to embrace every advantage that offers from the perfection of the work ; yet we do unhesitatingly condemn the wholesale method of warranting all operations, employed by many. It is not required by the intelligent patient who has confidence in the operator ; and no one should employ an operator if he has not full confidence in his ability.

There are many circumstances that we can not anticipate or control, which may affect the permanency of an operation, however well executed it may be ; and for these no dentist can in justice to himself become responsible, and no one who has a just appreciation of the subject, and desires to be just to himself and his patient, will do it.

In the great majority of cases no one can fill teeth, with an absolute assurance that their preservation will be permanent in the full sense of that term ; and he who gives an unlimited guarantee for the permanency of his work is either at fault in the matter, or ignorant. The proper course in all operations is to perform the work as well as possible ; at the same time giving the patient, if need be, a full explanation of the circumstances, that may modify the permanence of the work, and of the difficulties attending the operation, and of the probable efficiency of the work ; permitting the statement in no instance to exceed the probabilities of the case; under such circumstances if a filling is lost, no honest patient would demand the tooth refilled without charge.

When we have filled a tooth well, or as well as we can under the circumstances, and the filling gives way, and the patient desires it refilled, we do it, if we think there is a prospect of success, and charge the ordinary fee for it. If the surgeon amputates a limb and the patient dies, he collects his fee, and justly too. If the physician treats his patient and cures him, and he is sick again in a week and he treats him again, he collects both bills, even though the patient should die. If it is worth five dollars to fill a tooth once, it is worth five dollars to fill another just like it, or to fill the same one again; and there is no reason why the one should be done without a fee sooner than the other.

The question is often asked, "Dr., do you insure your plugs?" The invariable reply should be, "I do not, for if I make you a good operation, there is scarcely any comparison between the fee and the value of the operation to you, even if you are thereby enabled to retain a valuable tooth for a few years only."

Insurance upon dental operations is injustice to the patient as well as to the dentist. Nine-tenths of the patients who require and receive a guarantee upon their operations will manifest a total disregard for the cleanliness, and well-being of the teeth. They think their teeth are all "*fixed*," and "*insured*," and so they are safe without any further care on their part. But after a time the fillings give way and the teeth are lost, and the anathemas are heaped upon the head of the poor dentist, who was so unfortunate as to get into a false position.

The admitted necessity for a guarantee is a confession that the work is not well done. But the patient is quieted by the assurance that when this gives way, he will receive another of the same kind without charge. "Sweet consolation." This whole matter of dentists insuring their operations, is injustice to both the dentist and patient, and an imposition upon the profession. T.

